# Practical Physiology; for the Use of Schools and Families

**Published:** 1848-03

**Authors:** 


*Practical Physiology; for the Use of Schools and Families.* By EDWARD JARVIS, M. D. 12mo., pp. 368. Thomas, Cowperthwaite & Co. Philadelphia, 1848.

To popular treatises on diseases and their remedies we are on principle decidedly opposed; for the very sufficient reason, that all such must necessarily be very imperfect; and if they were otherwise, the amount of time and attention necessary for their proper comprehension would not be given to the subject by any but those who make it the business of their lives. On no other subject, according to our observation, do shallow draughts so intoxicate the brain, and certainly in nothing else is intoxication so dangerous. The half-instructed physician is always the most confident of his knowledge and his means, whilst the well-informed ponders at every step.

But the work before us is not liable to these objections. As its title sets forth, it is not designed for the professional reader. It is too brief, too general in its facts, and too limited in its scope. Such will of course drink at deeper fountains—as the books of Müller, Dunglison, or Carpenter. But for the class of readers referred to in the title, Dr. Jarvis's book seems to be of the right stamp. It supplies the amount of information which is necessary for the general scholar and the ordinary walks of life, without much argument or detail, and consequently without much consumption of time or labour of mind. There is in it no parade of cures or remedies but such as are hygienic, and these can be understood and acted on by every one. It is on these subjects that the author has indulged most in discussion; and although perhaps the most useful part of his production, it is on these topics that he has rendered himself most obnoxious to criticism.

The book is concisely written—sententious—sometimes quaint, but always intelligible.

